# Proteomic Profiling of the Microsomal Root Fraction: Discrimination of *Pisum sativum* L. Cultivars and Identification of Putative Root Growth Markers

**DOI:** 10.3390/proteomes5010008

**Published:** 2017-03-02

**Authors:** Claudia-Nicole Meisrimler, Stefanie Wienkoop, Sabine Lüthje

**Affiliations:** 1 Oxidative Stress and Plant Proteomics Group, Biocenter Klein Flottbek and Botanical Garden, University of Hamburg, Ohnhorststraße 18, D-22609 Hamburg, Germany; s.luthje@botanik.uni-hamburg.de; 2 Plant–Microbe Interactions, Department of Biology, Utrecht University, Padualaan 8, 3584 CH Utrecht, The Netherlands; 3 Deptartment of Ecogenomics and Systems Biology, University of Vienna, Althanstrasse 14, A-1090 Vienna, Austria; stefanie.wienkoop@univie.ac.at

**Keywords:** *Pisum sativum*, microsomes, cultivar comparison, root morphology

## Abstract

Legumes are a large and economically important family, containing a variety of crop plants. Alongside different cereals, some fruits, and tropical roots, a number of leguminosae evolved for millennia as crops with human society. One of these legumes is *Pisum sativum* L., the common garden pea. In the past, breeding has been largely selective on improved above-ground organs. However, parameters, such as root-growth, which determines acquisition of nutrients and water, have largely been underestimated. Although the genome of *P. sativum* is still not fully sequenced, multiple proteomic studies have been published on a variety of physiological aspects in the last years. The presented work focused on the connection between root length and the influence of the microsomal root proteome of four different pea cultivars after five days of germination (cultivar Vroege, Girl from the Rhineland, Kelvedon Wonder, and Blauwschokker). In total, 60 proteins were identified to have significantly differential abundances in the four cultivars. Root growth of five-days old seedlings and their microsomal proteome revealed a similar separation pattern, suggesting that cultivar-specific root growth performance is explained by differential membrane and ribosomal protein levels. Hence, we reveal and discuss several putative root growth protein markers possibly playing a key role for improved primary root growth breeding strategies.

## 1. Introduction

Pea cultivation developed together with human society for about 11,000 years [1]. Nowadays, *Pisum sativum* L. is one of the economical important legumes, including *Glycine max* (L.) Merr. (soybean), *Phaseolus* L. (bean), *Cicer arietinum* L. (chickpea), and *Arachis hypogaea* L. (peanut). In general, they are consumed in different forms. Peas are a rich source for proteins (23%–25%) and essential amino acids, complex carbohydrates, and minerals, like iron, calcium, and potassium. In the most recent trends, pea seedlings are used to produce protein concentrates for protein powders as an alternative to dairy-derived whey proteins or glycine proteins. According to FAOSTAT data world production of the garden pea has more than quadrupled in the last 30 years [2]. The pea has been investigated as a model with respect to several physiological aspects and, interestingly enough, mapping the pea genome has lagged behind other crops because it has such a large and complex genome [3]. Aside from this, databases with information on pea genomic markers, genetic maps, quantitative trait loci (QTLs), and others are available on the coolseasonfoodlegume database, which is also usable for proteomic analysis [4]. 

Many proteomic studies concerning abiotic and biotic stress factors have been published in recent years [5,6,7]. Proteomic approaches in cultivar analysis, especially for orphan species, are an alternative to transcriptomics or genomics, and directly examine alterations in protein profiles. Protein quantitative trait loci (PQTLs), as used in medical applications [8], might be an alternative for cultivar analysis in the future and be of importance for production of stress tolerant and resistant pea cultivars. The use of proteomic approaches to analyse pea increased rigorously in recent years [9,10,11,12]. Generally, pea cultivars differ clearly in morphological and agronomic traits, in physiological and biochemical characteristics, and in their genomic structure. However, the proteins and genes responsible for these differences remain poorly characterized. Therefore, identifying cultivar-dependent differences of protein levels and composition are of interest for different agricultural applications. Roots are the major site of nutrient and water uptake and transport processes, therefore, they are of major interest for improved cultivars regarding mineral shortages, waterlogging, heavy metal pollution, and others. Furthermore, root growth is an important component of plant growth, but has received little attention by plant breeders due to difficulties associated with root observation. Improved root-related traits are likely to improve pea production, as well [12]. In the past, root length and root density have been observed for different abiotic stress factors, like phosphate starvation [13]. Root density is a far more complex trait than root length, making it more complicated to link proteomic datasets to this trait; therefore, we decided to focus first on root length in seedlings, rather than root density at later developmental stages. 

The microsomal fraction, which consists mainly of membrane and ribosomal proteins, was frequently used in the past for proteomic approaches [14]. They contain an enriched amount of membrane proteins, relevant in root elongation growth, hormone receptors (e.g., ABP1), signal transduction, transport, and others, making them specifically interesting for root growth-related traits [15]. Changes in membrane proteins can often only be followed by proteomic approaches or Western blotting due to a mismatch of the transcriptional level to protein amounts [16]. In the present study we used four pea cultivars (*Pisum sativum* L. var. *axiphium* cultivar Vroege (Vroege), *Pisum sativum* L. var. *sativum* cultivar Girl from the Rhineland (GftR), *Pisum sativum* L. var. *medullare* cultivar Kelvedon Wonder (Kelvedon), and *Pisum sativum* L. var. *sativum* cultivar Blauwschokker (Blauwschokker) of different phenotypes. All four cultivars are traditionally cultivated peas in Europe, therefore, they are of interest for a broad group of applicants. According to the distributor their size is decreasing in the order Vroege (80 cm) > Blauwschokker (60–80 cm) > Kelvedon (50–60 cm) > Girl from the Rhineland (20–30 cm). The latter cultivar is a dwarf mutant with a defect in gibberellic acid biosynthesis. Cultivar specific protein profiles and protein abundance will be investigated in microsomal fractions. The correlation between root length and “marker proteins” will be discussed.

## 2. Materials and Methods 

### 2.1. Plant Material

The four different European dish peas (cultivar Vroege, cultivar Girl from the Rhineland, cultivar Kelvedon Wonder, cultivar Blauwschokker; all cultivars were purchased from Sperli, Everswinkel, Germany) were cultivated for five days on paper. Seeds were washed with circulating tap water for 30 min, submerged in 1% H_2_O_2_ for 10 min and washed with 50% ethanol for 2 min. After each disinfection step, seeds were rinsed with tap water. Germination was accomplished for five days in the dark at 26 °C.

### 2.2. Microsomal Enrichment 

Roots (50 g fresh weight, 120–160 plants) were harvested and proteins extracted as described in Meisrimler et al. (2011) [6]. Membrane proteins were separated from the cell debris and soluble proteins by differential centrifugation (10 min at 10,000× *g* and 30 min at 50,000× *g*). 

### 2.3. Washing Membranes

The microsomal-enriched fraction was washed for 45 min at 4 °C with 20 mM Tris-HCl pH 7.6, 150 mM KCl, 150 mM sucrose, 0.01% Triton X-100 and 1 mM ethylenediaminetetraacetic acid (EDTA) to remove peripheral proteins and adsorbed soluble proteins as described in Meisrimler et al. [17]. Protein amounts were quantified as described by Bradford [18] in the presence of 0.01% Triton X-100 using bovine serum albumin as the standard.

### 2.4. Sample Preparation for Mass Spectrometry 

Three independent microsomal preparations (for each cultivar) were used for the proteomic approach. Proteins were precipitated in chloroform/methanol 1:4 at −20 °C overnight. Following centrifugation for 20 min at 12,000× *g* and 4 °C, the pellet was washed with methanol three times. The pellet was resuspended in 200 mM NH_4_HCO_3_ (Merck, Darmstadt, Germany) pH 8.5 containing 8 M urea and 10% acetonitrile. One hundred micrograms of protein for each replicate were reduced, alkylated, and pre-digested with LysC (Roche, Mannheim, Germany) for 6 h at 37 °C. Afterwards, the solution was diluted to 50 mM NH_4_HCO_3_ pH 8.5, 2 M urea and 10% acetonitrile for digestion with trypsin beads (Applied Biosystems, Darmstad, Germany), which was carried out for 16 h at 37 °C. After removal of the beads by centrifugation, the supernatant was desalted using C_18_-columns, eluted with acetonitrile, and dried in a Speed Vac (Savant ISS110, Thermo Scientific, Bremen, Germany). Peptides were dissolved in 5% acetonitrile and 0.1% formic acid, and centrifuged again for 20 min at 12,000× *g* prior to LC-MS/MS analysis.

### 2.5. Mass Spectrometry

MS analysis was performed using an LTQ/Orbitrap XL (Thermo Scientific, Bremen, Germany) at a resolution of 30,000×, coupled to a 1D nano ultra Eksigent HPLC-System (Axel Semrau, Sprockhövel, Germany). Half a microliter of protein digest per sample was loaded on a C_18_ column (75 µm internal diameter, 15 cm × 0.1 mm, 2.7 µm; Sigma-Aldrich, Vienna, Austria) which was used to separate the peptides. The mobile phases A (0.1% formic acid, 5% acetonitrile) and B (0.1% formic acid, 80% acetonitrile) were used to form a gradient of 5%–60% acetonitrile in 60 min. The flow rate was 400 nL·min^−1^.

Proteins were identified using the SEQUEST algorithm and the Proteome Discoverer (v 1.3, Thermo Scientific Inc.) to search MS data against an in-house fasta-file. The fasta-file was created as described previously [19] from downloads of different *Pisum sativum* sequence databases, fused from UniProt Uniref100 and a six frame translation of the Pisum_sativum_v1_Contig1005 (contig) *Pisum sativum* DB http://www.coolseasonfoodlegume.org/ID56124, and PHVGI_Prot_rel4DFCI DB.

In silico peptide lists were generated with the following settings: trypsin as the digestion enzyme and a maximum of three missed cleavages. Mass tolerance was set to 5 ppm for precursor ions and 0.8 Da for fragment ions. Additionally, a decoy database containing reversed sequences was used to estimate the false discovery rate (FDR). Only high confidence (FDR ≤ 0.01%) peptide identifications with a minimum XCorr of 2.0 and proteins with at least two distinct peptides were considered. The data matrix of the Proteome Discoverer (ThermoFisher Scientific, Darmstadt, Germany), containing spectral count information was used. Missing values were replaced by 0.5 (half of minimal value). For statistical analysis, a Student’s *t*-test was carried out (*p* ≤ 0.05 for significance). For multivariate statistics (PCA), the MATLAB tool Covain was used [20]. A functional annotation was assigned using a Mapman mapping file, created using Mercator [21] on the basis of the in-house fasta-file assembled according to [22].

### 2.6. Targeted Protein Marker Evaluation

Several prototypic peptides of four mitochondrial membrane proteins (Supplemental Table S2) were selected for a targeted summed-intensity extraction using our ProtMax software (University of Vienna, Vienna, Austria) [23]. 

## 3. Results

### 3.1. Morphological Changes

**Figure 1:**
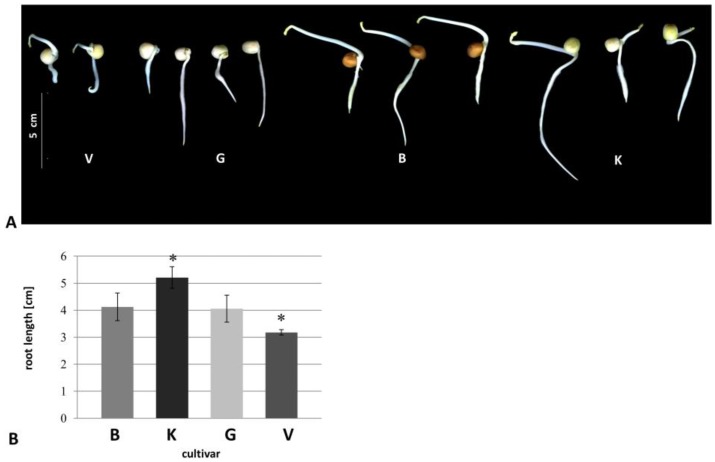
Five-days old seedlings. (**A**) Seedlings at day five; (**B**) Root length at day five. ■ Cultivar Blauwschokker (B), ■ cultivar Kelvedon (K), ■ cultivar GftR (G), ■ cultivar Vroege (V). Significant values *p* ≤ 0.05 were marked with an asterisk. *n* ≥ 20.

All cultivars with typical phenotypes were shown in Figure 1A. Vroege showed significantly shorter roots than the other cultivars (Figure 1B), whereas cultivar Kelvedon exhibited the longest roots (Figure 1B). In general, even cultivar Kelvedon showed, significantly, the longest roots, a high variation in root length was observed in each cultivar, except for Vroege.

### 3.2. Proteome Analysis

#### 3.2.1. General Overview

**Figure 2:**
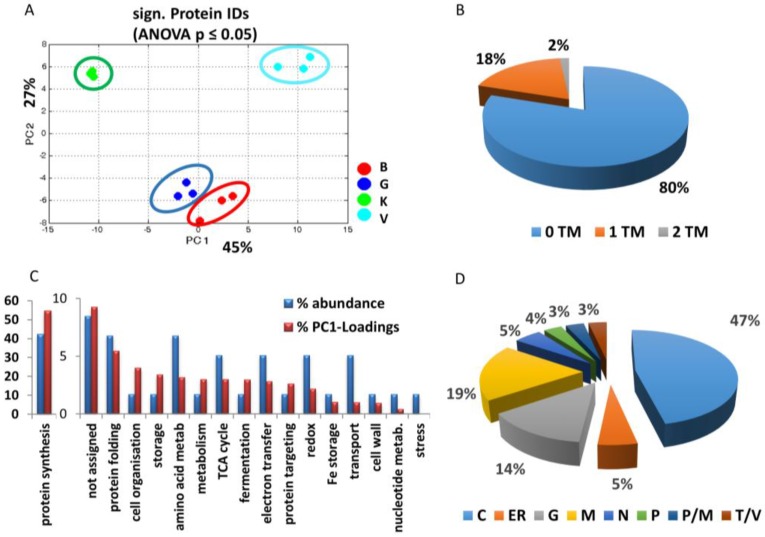
Comparison of the microsomal enriched and significantly different proteins across roots of four pea cultivars by shotgun LC-MS. MS analyses were performed using an LTQ/Orbitrap XL and significantly different (*t*-test, *p* ≤ 0.05) proteins were analysed in detail by PCA (**A**): B, Blauwschokker (red); G, GftR (blue); K, Kelvedon (green); V, Vroege (cyan). Significant proteins shown in Table 1 were analysed for transmembrane (TM) domains using HMMTOP (**B**), percentage of proteins corresponding to the functional groups in relation to their PC1 loadings (**C**), and cellular localisation of proteins: M; mitochondria; P; plastid; G; golgi; N; nucleus; C; cytosol; T; tonoplast; V; vesicles (**D**).

In total, 241 root proteins of a microsomal enrichment were identified from the LysC/tryptic in-solution digest by LC-MS (Supplemental Table S1). Among these were the standard marker of outer mitochondrial membrane (VDAC), inner mitochondrial membrane (cytochrome c oxidase), and endoplasmatic reticulum (KDEL-containing protein disulfide-isomerase). As shown in Supplemental Table S2, abundance of mitochondrial markers was similar in all cultivars, whereas that of the ER marker decreased from GftR > Vroege > Kelvedon > Blauwschokker. The levels of 60 proteins were changed differentially and statistically significant in the four cultivars at this developmental stage of the root (Table 1). PC1 (explaining 45% of the discrimination), separated membrane proteins of the cultivars that seemed related to root growth; from Kelvedon, with the longest root growth, to a clustering of Blauwschokker and GftR, and then Vroege, with the smallest growth (Figure 1A and Figure 2A).

The smallest number (20) of significantly-changed proteins was found between GftR and Kelvedon, and a large number (36) for e.g., Kelvedon against Vroege (Table 1). The differences between the latter cultivars were also underlined by the most distinct changes in the protein ratio.

Overall, 20% of the significantly-changed proteins shown in Table 1 had a transmembrane region (Figure 2B). Most proteins identified to be of differential abundance were related to protein synthesis (42%), protein folding (7%) and amino acid metabolism (7%), where this percentage is called % abundance (Figure 2C). In addition, 8.5% of the proteins with a significant difference between the cultivars could not be assigned to any specific function (Figure 2C). All significantly-different proteins were analysed for their subcellular localisation, using information for the corresponding protein entry in NCBI or Uniprot. If no data about localisation were available, PlantmPLOC was used to compute localisation. The highest amount of proteins identified appeared to be cytosolic origin (47%), followed by mitochondria (19%), and golgi (14%) (Figure 2D).

#### 3.2.2. Cultivar Specific Differences—Proteins with the Highest Separation Impact on PC1 

The largest functional group of significantly-changed protein levels between the cultivars was protein synthesis, which also showed the highest percentage of PC1 loadings (~55%) (Figure 2C). In total, six proteins of the 60S ribosome and twelve of the 40S ribosome were identified to be differentially abundant in the cultivars. All ribosomal proteins showed the significantly highest abundance in cultivar Vroege. 

In contrast, the channel protein TOM40 homolog1—categorized to protein targeting—was clearly of highest abundance in Kelvedon compared to the other cultivars (up to 16.6-fold). This protein is most likely to be localized in the mitochondria outer membrane (Table 1).

Other protein groups that show a higher percentage of PC1 loadings (high impact on separation) compared to their abundance (in terms of numbers of proteins) were assigned to cell organisation, storage, metabolism, fermentation, as well as the group of unassigned proteins (Figure 2C).

For instance, convicilin, a major storage protein of pea, was eleven to 22-fold higher in cultivar Vroege. Furthermore, metabolism-related protein dihydrolipoamide dehydrogenase, the fermentation-related protein ADH family 2 member B7 protein and, most significantly, regarding both abundance and PC1 loading, annexin-like protein RJ4, located to the nucleus membrane, were mainly increased in Vroege (Table 1). 

All other groups, such as amino acid metabolism, redox and electron transfer, cell wall and transport, showed only a low percentage on the impact of PC1 loadings, which was also comparably lower than their abundances and, thus, less likely related to root growth.

## 4. Discussion

Results once again displayed the difficulty of proteomic approaches for membrane proteins. Only 20% of the significantly changed proteins had a transmembrane region (Figure 2B) and a major accumulation of ribosomes was observed. This might be due to cytosolic contamination with ribosomal proteins, or it might be caused by the interaction with the (rough) endoplasmic reticulum, as previously discussed [29]. Other reasons might be the interaction with membrane proteins, e.g., during folding processes. Ribosomal proteins represent higher abundant proteins and are usually easily identified in microsomal fractions, which has been known for a relatively long time and was not only restricted to plant samples [17,30,31,32,33]. In general, it has been shown that a high plasticity exists in ribosomal protein regulation that is part of adaptive processes to differential stress or developmental aspects [32,33]. Furthermore, the chosen centrifugation speeds for the preparation of the microsomal enrichment, which were lower than for the usual microsomal preparations, might have resulted in a higher amount of cellular compartments, which might also present a lower coverage of membrane proteins. However, this does not influence the results on the proteins and how they relate to the root phenotype.

In this study, we found that morphological root phenotypes of four diverse pea cultivars are likely to be explained by their differential microsomal root proteomes. Vroege had the significantly shortest, and Kelvedon longest roots, respectively, and compared to the other cultivars (Figure 1B). Morphology of these four cultivars have been observed in earlier studies for older plants grown in hydroponics, showing that root length was only slightly different between cultivars, with Blauwschokker and Kelvedon exhibiting the longest roots [17]. If the differences in root length are due to different growth or differentiation, as well as the aspect of different hormones, should be investigated in the future.

Cultivar Kelvedon has previously been observed to be highly efficient in iron-deficient and high pH conditions [17,34]. Kelvedon is also resistant to pea wilt and tolerant to downy mildew (in reference to different breeders e.g., Tompson and Morgan, and the Royal Horticulture Society, www.rhs.org.uk). The latest data also suggest a higher Cd tolerance of Kelvedon [35]. Furthermore, a significant increase of Cd in roots, but not in leaves, was observed due to Cd stress. To date, cultivar Kelvedon has been shown in multiple studies to be superior in heavy metal-related stresses to other cultivars, and it would be interesting to see how this traditional cultivar is affected by other stresses, e.g., cold and water stress, and the reason for it. This will be an important step to breed more resistant cultivars in the future. In contrast, earlier studies have described Vroege to be relatively sensitive to iron deficiency conditions [17].

The generally increased protein levels, along with the root growth characteristics found at day five after germination, described above, might display a connection between increased stress susceptibility and a short root morphology. It is well known that enhanced root length can increase drought stress tolerance [36,37]. Determining if this trait is also involved in the enhancement of other stresses needs further investigation.

Altogether, 60 root microsomal proteins were found differentially abundant in the four cultivars after five days of germination (Supplemental Tables 1 and 2). The PCA analysis underpinned that several of these proteins might be involved in the different root phenotypes of the four cultivars (Figure 2A). Nevertheless, it remains unclear whether the difference was cause or the consequence of the developmental disparities.

The discussion is focused on those proteins appearing to impact most on the separation between the root phenotypes, which are potentially good markers. Interestingly, annexin-like protein RJ4 tagged to the nucleus showed the highest impact on the differentiation between the cultivars’ root phenotypes. Annexins and annexin-like proteins are regularly found in proteomic analysis of membrane fractions [38,39]. They have a function in calcium-dependent phospholipid binding and are involved in root and nodule development [39,40,41]. Baucher et al. [42] described a tobacco annexin (Ntann 12) mainly localized in the nucleus of the root cells. The accumulation of Ntann 12 was strongly linked to auxin transport and signalling, which is also well known to play an essential role during root development [43,44]. In case of high auxin concentrations, root elongation is inhibited [45]. In addition, auxin accumulation in root cells was described to induce annexin Ntann12 [42]. In this study, annexin accumulation, therefore, implies that auxin levels were also increased in roots of cultivar Vroege. Hence, annexin-like protein RJ4 found in our study seems to be a good marker for hampered root development. Its accumulation might also reflect an early stage of development. The latter option may be aided by the finding of another putative marker, convicilin, solely enhanced in cultivar Vroege. Convicilin is a seed storage protein involved in seed development that was found earlier and described for pea [46]. It has been shown before that, in the pea embryonic axis, the storage proteins, including convicilin, changed during germination [11]. Its accumulation in cultivar Vroege underlines its earlier stage of seed development. Hence, the combination of the two putative marker proteins might be important to distinguish between developmental stages and root growth performance. However, this needs to be further analysed in the future.

As another interesting candidate, endoplasmin is a molecular chaperone of the heat-shock protein 90 class located in the endoplasmic reticulum [47], which plays a role in folding and is a highly regulated protein family. Due to the importance of endoplasmins for different stress conditions and developmental stages, these proteins have been found often regulatory relevant in proteomic studies [48,49]. Here, the strong induction of this protein (contig17941), together with several ribosomal proteins, implies an impairment in protein synthesis pathways leading to reduced root growth. 

In contrast to the above-discussed putative markers for reduced root growth, our data provide evidence for a mitochondrial marker, TOM40 homolog 1, indicating enhanced root development. TOM40 is a central component of a hydrophilic channel of the mitochondrial outer membrane, the so-called translocase of the outer membrane (TOM), functioning as a protein import pore [50]. Thus, it is proposed that TOMs are directly linked with precursor proteins to participate in early stages of mitochondrial protein import. Mitochondria play an important role in energy-, redox-, and metabolic homeostasis, and have been shown to be integrated with plant growth regulation. Most mitochondrial mutants revealed reduced growth rates and have a short root phenotype [51]. Even though it is not clear how the TOM machinery might control the root development, elevated TOM40 levels appeared to be associated with increased root growth. This is also supported by the fact that other mitochondrial membrane markers were not significantly changed between the cultivars.

Several other proteins that significantly differentiate between the cultivars, such as proteins involved in transport, amino acids and redox-related proteins do not show an impact on the separation between the root phenotypes, but may play a role in other developmental regulations that cannot be unravelled in this study. 

## 5. Conclusions

Summarizing, proteomic approaches have the potential to be used as alternative techniques to QTL mapping. Here, our shotgun proteomics approach enabled distinguishing different pea cultivars according to their root microsomal proteome linked to their root phenotypes. In contrast to genes, proteins represent the last instance for molecular reactions and the proteomic approach are complementary in finding implications between phenotypes, genotypes, and specific trades breeders are looking for. The analysed pea cultivars revealed microsomal proteins with high impacts on root development at the seedling stage possibly important for many other plants. We found the mitochondrial channel protein TOM40 to be most promising as a putative marker for improved root growth, which will be further investigated in the future. In contrast, the accumulation of an annexin-like protein RJ4 and convicilin seemed to be a potential marker associated with shortened root growth.

## Figures and Tables

**Table 1:** Significantly different proteins in the four cultivars identified by LC-MS. The heat map table shows all identified proteins that changed significantly in at least one of the cultivars. Each measurement consisted of three biological replicates and each biological replicate was a pool of 100 plants to keep variation of individuals low. Significant changes in ratios (Student’s *t*-test< 0.05; ratio <0.5 or ratio >2.0) are marked in bold. Decreased values are marked in green; increased values are marked in red. Numbering is given in the first column. Accession numbers and protein description correspond to the database used for identification. TM, transmembrane domains: TM1 was computed with TMHMM [24] and TM2 was computed with HMMTOP [25]. Cellular function of the proteins; based on MapMan and manual validation with InterProScan. Localization (Loc) is based on Uniprot or NCBI information and, if localization was unknown, marked with an asterisk. PlantMPloc was used for prediction [26,27,28]; localization: M; mitochondria; P; plastid; G; golgi; N; nucleus; C; cytosol; T; tonoplast; V; vesicles. Column 6–9, show the average spectral counts (SC). Columns 10–15, show the ratios for all possible cultivar comparisons. In the last column, PC1 loading is shown. Amount of significant fold changes: blue shades, decrease; red shades, increase. Statistical significant data for *p* ≤ 0.05 are shown in bold. Abbreviations for the cultivars were chosen as follows: B; Blauwschokker; V; Vroege; G; Girl from the Rheinland, GftR; K; Kelvedon Wonder. Significant fold changes: blue shades, decrease; red shades, increase.

Accession	Description	TM1	TM2	Function			SC			Ratio						PC1 loadings
					loc	B	V	G	K	V/K	B/K	G/K	V/G	V/B	G/B	
contig12248	Annexin-like protein RJ4	0	0	cell organisation	N *	5.7	18.7	3.7	0.5	**37.3**	**11.3**	**7.3**	**5.1**	**3.3**	0.7	0.2490
p.sativum_wa1_contig21697	60S ribosomal protein L4-1	0	0	protein synthesis	C	10.3	15.3	8.7	0.5	**30.7**	**20.7**	**17.3**	**1.8**	**1.5**	**0.8**	0.2379
contig10741	Convicilin	0	0	storage	G *	1.0	11.7	0.5	0.5	**23.3**	2.0	1.0	**23.3**	**11.1**	0.5	0.2130
p.sativum_wa1_contig28348	40S ribosomal protein S8	0	0	protein synthesis	C	3.2	8.0	1.7	0.5	**16.0**	6.3	3.3	**4.8**	**2.5**	0.5	0.2017
p.sativum_wa1_contig16856	60S acidic ribosomal protein P2-2	0	0	protein synthesis	C	2.0	10.7	4.0	0.5	**21.3**	4.0	**8.0**	**2.7**	**5.3**	2.0	0.1961
contig17941	Endoplasmin homolog	0	0	protein folding	ER	2.5	9.0	6.0	0.5	**18.0**	5.0	**12.0**	1.5	**3.6**	**2.5**	0.1904
9955324 pdb	Dihydrolipoamide dehydrogenase	0	0	metabolism	M*	1.3	8.0	0.5	0.5	**16.0**	2.7	1.0	**16.0**	**5.9**	0.4	0.1878
Pisum_sativum_v2_Contig4072	ADH family 2 member B7	0	1	fermentation	M	12.3	10.3	5.7	1.0	**10.3**	**12.3**	**5.7**	1.8	0.8	**0.5**	0.1850
contig02118	60S ribosomal protein L13-1	0	0	protein synthesis	C	1.7	6.7	0.5	0.5	**13.3**	3.3	1.0	**13.3**	**4.0**	0.3	0.1830
p.sativum_wa1_contig25888	40S ribosomal protein S4-2	0	0	protein synthesis	C	3.0	6.0	2.2	0.5	**12.0**	**6.0**	4.3	**2.8**	**2.0**	0.7	0.1766
Contig3080	40S ribosomal protein S17-4	0	0	protein synthesis	C	4.0	5.0	0.5	0.5	**10.0**	**8.0**	1.0	**10.0**	1.3	**0.1**	0.1763
EX568861	40S ribosomal protein S17	0	0	protein synthesis	C	4.0	5.0	0.5	0.5	**10.0**	**8.0**	1.0	**10.0**	1.3	**0.1**	0.1763
GH720005	60S ribosomal protein L9	0	0	protein synthesis	C	5.0	6.3	5.0	0.5	**12.7**	**10.0**	**10.0**	1.3	1.3	1.0	0.1755
p.sativum_wa1_contig17528	60S ribosomal protein L13-1	0	0	protein synthesis	C	1.7	5.3	1.3	0.5	**10.7**	3.3	2.7	**4.0**	3.2	0.8	0.1642
Pisum_sativum_v2_Contig5665	60S ribosomal protein L4	0	0	protein synthesis	C	2.5	5.3	1.3	0.5	**10.7**	5.0	2.7	**4.0**	2.1	0.5	0.1621
p.sativum_wa1_contig24787	40S ribosomal protein S2-4	0	0	protein synthesis	C	1.5	5.3	0.5	0.5	**10.7**	3.0	1.0	**10.7**	**3.6**	0.3	0.1604
contig13719	40S ribosomal protein SA	0	0	protein synthesis	C	0.5	6.0	0.5	0.5	**12.0**	1.0	1.0	**12.0**	**12.5**	1.0	0.1585
p.sativum_wa1_contig21882	60S ribosomal protein L4-1	0	0	protein synthesis	C	2.2	5.0	1.3	0.5	**10.0**	4.3	2.7	**3.8**	2.3	0.6	0.1570
p.sativum_wa1_contig07673	40S ribosomal protein S15-4	0	0	protein synthesis	C	5.0	4.7	6.0	0.5	**9.3**	**10.0**	**12.0**	0.8	0.9	1.3	0.1541
p.sativum_wa1_contig22133	40S ribosomal protein SA	0	0	protein synthesis	C	0.5	5.3	0.5	0.5	**10.7**	1.0	1.0	**10.7**	**11.1**	1.0	0.1517
ACU20293.1	40S ribosomal protein S30	0	0	protein synthesis	C	4.3	4.3	4.7	0.5	**8.7**	**8.7**	**9.3**	0.9	1.0	1.1	0.1486
ACJ85955.1	60S ribosomal L12-like protein	0	0	protein synthesis	C	1.3	4.3	1.5	0.5	**8.7**	2.7	3.0	**2.9**	3.2	1.1	0.1429
BAB40231.1	S-type apyrase	0	1	not assigned	N*	8.0	3.3	8.0	0.5	**6.7**	**16.0**	**16.0**	0.4	**0.4**	1.0	0.1345
p.sativum_wa1_contig03572	40S ribosomal protein S3a	0	0	protein synthesis	C	0.5	3.7	0.5	0.5	**7.3**	1.0	1.0	**7.3**	**7.1**	1.0	0.1274
contig09310	Uncharacterized protein At5g10860	0	0	not assigned	M	1.7	2.7	0.5	0.5	**5.3**	3.3	1.0	**5.3**	1.6	0.3	0.1241
Pisum_sativum_v2_Contig1288	40S ribosomal protein S3a	0	0	protein synthesis	C	0.5	3.3	0.5	0.5	**6.7**	1.0	1.0	**6.7**	**6.7**	1.0	0.1209
Pisum_sativum_v2_Contig4787	Protein disulfide isomerase-like 1-4	0	1	redox	ER	4.7	6.0	7.7	1.7	3.6	2.8	**4.6**	0.8	1.3	1.7	0.1198
Pisum_sativum_v2_Contig857	40S ribosomal protein S18	0	0	protein synthesis	C	0.5	3.0	0.5	0.5	**6.0**	1.0	1.0	**6.0**	**5.9**	1.0	0.1132
p.sativum_wa1_contig04121	LOX homol. domain-containing protein 1	0	0	not assigned	G*	2.3	2.7	3.7	0.5	**5.3**	**4.7**	**7.3**	0.7	1.1	1.7	0.1119
Pisum_sativum_v2_Contig5583	Succinyl-CoA ligase sub. b	0	1	TCA cycle	G*	0.5	5.3	0.5	1.3	**4.0**	0.4	0.4	**10.7**	**11.1**	1.0	0.1109
GH720468	40S ribosomal protein S25-2	0	0	protein synthesis	C	4.3	3.5	3.3	0.5	7.0	**8.7**	**6.7**	1.1	0.8	0.8	0.1089
Contig386	40S ribosomal protein S14	0	0	protein synthesis	C	11.3	15.0	12.0	4.7	**3.2**	**2.4**	**2.6**	1.3	**1.3**	1.1	0.0823
GH720846	60S ribosomal protein L22-2	0	0	protein synthesis	C	3.0	1.8	1.5	0.5	3.7	**6.0**	3.0	1.2	0.6	**0.5**	0.0781
contig09315	Cysteine synthase	0	0	amino acid metab	G	16.7	15.7	6.7	6.3	**2.5**	**2.6**	1.1	**2.4**	0.9	**0.4**	0.0702
AAB24082.1	ferritin	0	0	Fe storage	P	4.3	8.7	4.3	3.3	**2.6**	1.3	1.3	2.0	2.0	1.0	0.0643
contig21497	Cysteine synthase	0	0	amino acid metab.	P/M	18.7	16.0	7.7	7.3	**2.2**	**2.5**	1.0	**2.1**	0.9	**0.4**	0.0628
Pisum_sativum_v2_Contig4779	UDP-glucuronic acid decarboxylase 1	1	1	cell wall	G/N *	0.5	1.8	3.7	0.5	3.7	1.0	**7.3**	0.5	3.7	**10.0**	0.0584
contig20699	NADH-Q oxidoreductase 40 kDa sub.	0	0	electron transfer	M	3.0	3.3	0.5	2.5	1.3	1.2	0.2	**6.7**	1.1	**0.2**	0.0532
p.sativum_wa1_contig18574	Chaperonin CPN60-2	0	0	protein folding	M	17.7	18.7	7.3	10.3	1.8	1.7	0.7	**2.5**	1.1	**0.4**	0.0515
contig22493	Cysteine synthase	0	0	amino acid metab	G	18.7	10.0	5.3	6.3	1.6	**2.9**	0.8	1.9	0.5	**0.3**	0.0472
Pisum_sativum_v2_Contig6323	component of 2-oxoglutarate dehydrogenase	0	1	TCA cycle	G*	5.0	4.7	1.0	3.3	1.4	1.5	0.3	**4.7**	0.9	**0.2**	0.0455
p.sativum_wa1_contig18562	Chaperonin CPN60-2	0	0	protein folding	M	14.3	17.7	5.3	11.0	1.6	1.3	**0.5**	**3.3**	1.2	**0.4**	0.0439
Pisum_sativum_v2_Contig1332	Probable arginase	1	1	amino acid metab.	G	4.3	8.0	11.3	5.3	1.5	0.8	**2.1**	**0.7**	1.9	**2.5**	0.0191
189095946 pdb	Mitochondrial Type Ii Peroxiredoxin	0	0	redox	M	0.5	5.7	0.5	4.0	1.4	**0.1**	0.1	11.3	**11.1**	1.0	0.0130
p.sativum_wa1_contig02960	Elongation factor 1-alpha	0	0	protein synthesis	C	1.3	4.3	5.3	3.3	1.3	**0.4**	1.6	0.8	**3.2**	**3.3**	0.0026
P37900.585272 sp	HSP70	0	0	stress	M	0.5	6.0	0.5	5.3	1.1	**0.1**	**0.1**	12.0	**12.5**	1.0	-0.0022
Pisum_sativum_v2_Contig5744	Probable protein disulfide-isomerase A6	0	1	redox	ER	12.7	25.7	18.3	25.3	1.0	**0.5**	**0.7**	1.4	**2.0**	**1.4**	-0.0040
Pisum_sativum_v2_Contig5499	NADH-Q oxidoreductase subunit	0	1	electron transfer	G*	5.0	7.7	2.5	9.3	0.8	**0.5**	0.3	**3.1**	**1.5**	**0.5**	-0.0090
G9JKP3	Plastid OEP 16.2	0	2	transport	P	0.5	5.7	2.5	5.7	1.0	**0.1**	0.4	2.3	**11.1**	5.0	-0.0138
CBD35496.1	Elongation factor 1-alpha	0	0	protein synthesis	C	0.5	3.7	4.0	3.3	1.1	**0.2**	1.2	0.9	**7.1**	**10.0**	-0.0179
p.sativum_wa1_contig06534	V-type proton ATPase 116 kDa sub.a 1	0	0	transport	T/V	3.0	0.5	0.5	1.3	0.4	2.3	0.4	1.0	**0.2**	**0.2**	-0.0201
p.sativum_wa1_contig17185	Elongation factor 1-alpha	0	0	protein synthesis	C	0.5	3.0	2.5	3.0	1.0	**0.2**	0.8	1.2	**5.9**	**5.0**	-0.0217
56554368 pdb	Nucleoside diphosphate kinase	0	1	not assigned	M	1.0	4.0	2.5	5.0	0.8	**0.2**	0.5	1.6	**4.0**	**2.5**	-0.0246
p.sativum_wa1_contig12891	Adenylate kinase B	0	0	nucleotide metab.	C	0.5	8.3	4.0	8.0	1.0	**0.1**	0.5	**2.1**	**16.7**	**10.0**	-0.0259
p.sativum_wa1_contig19033	V-type proton ATPase subunit E	0	0	transport	T/V	8.0	2.7	6.0	4.7	0.6	**1.7**	1.3	0.4	**0.3**	0.8	-0.0281
contig10734	SDH flavoprotein subunit 1	0	0	TCA cycle	M	2.0	6.7	1.7	10.0	0.7	**0.2**	**0.2**	**4.0**	3.3	0.8	-0.0313
contig20456	HSP 70 kDa	0	0	protein folding	M	0.5	3.5	0.5	4.3	0.8	**0.1**	**0.1**	7.0	7.1	1.0	-0.0558
p.sativum_wa1_contig16648	ferredoxin	0	0	electron transfer	P/M	4.3	1.0	3.3	5.0	**0.2**	0.9	0.7	0.3	**0.2**	0.8	-0.1138
Pisum_sativum_v2_Contig2233	TOM40 homolog 1	1	1	protein targeting	M	0.5	1.3	0.5	8.3	**0.2**	**0.1**	**0.1**	2.7	2.6	1.0	-0.1632
Q43877	Duplicate protein - HMG-I/Y	0	0	not assigned	N	0.5	0.5	6.0	5.3	**0.1**	**0.1**	1.1	0.1	1.0	**10.0**	-0.1842
							**Significant fold change**			**36**	**31**	**20**	**31**	**29**	**23**	
										**41**	**46**	**36**	**42**	**37**	**29**	

## Supplementary Materials

Supplementary materials are available online.
